# Hybrid Nanofiller-Enhanced Carbon Fiber-Reinforced Polymer Composites (CFRP) for Lightning Strike Protection (LSP)

**DOI:** 10.1021/acsomega.4c03272

**Published:** 2024-08-09

**Authors:** Matheus Mendes de Oliveira, Linnea Runqvist, Thirza Poot, Kajsa Uvdal, Danilo Justino Carastan, Linnea Selegård

**Affiliations:** † Center for Engineering, Modeling and Applied Social Sciences, Federal University of ABC, Santo Andre, São Paulo09210580, Brazil; ‡ Saab AB, Linköping SE-581 88, Sweden; § Division of Molecular Surface Physics and Nanoscience, Department of Physics, Chemistry and Biology (IFM), Linköping University, Linköping SE-581 83, Sweden

## Abstract

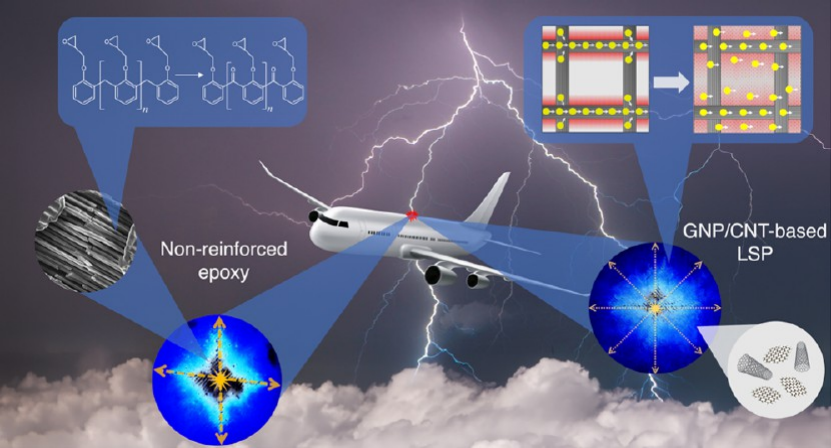

The aviation industry relies on lightweight carbon fiber-reinforced polymers (CFRP) for fuel efficiency, which necessitates lightning strike protection (LSP) and electromagnetic shielding due to their electrical insulating characteristics. Traditional metallic meshes used for LSP are heavy and corrosion-prone, prompting the exploration of alternatives. This research showcases CFRP nanocomposites with enhanced LSP properties through the incorporation of graphene nanoplatelets (GNPs) and carbon nanotubes (CNTs). While the enhanced conductivity in the nanofilled epoxy matrix did not impact the overall conductivity of CFRP panels, a significant damage reduction was observed after simulated lightning strike tests. Similar approaches in the literature have also noted this discrepancy, but no attempts to reconcile it have been made. This work provides a framework to explain the damage reduction mechanism while accounting for the modest conductivity improvements in the nanoreinforced CFRPs. Additionally, a simple, nondestructive method to assess surface resin degradation after a lightning strike test is proposed, based on the fluorescence of diphenyl ketones. The discussion is supported by electrical conductivity measurements, damage pattern evaluation using the proposed UV-illumination method, ATR-FTIR, and scanning electron microscopy analysis pre- and postlightning strike simulation.

## 1 Introduction

The global emission of greenhouse gases has been recognized as one of the largest environmental threats to our society and sustainable growth. In this context, aviation accounted for 3.8% of the total CO_2_ emissions in the European Union in 2017^1^ and 2.5% of global CO_2_ emissions in 2018,^2^ making it the second largest emitter in the transport sector after road transport. Reduction of aircraft structural weight is identified as one of the routes for a successful decrease in fuel consumption and thereby CO_2_ emission. In the modern generations of aircraft, metal structures were replaced by lighter carbon fiber-reinforced polymer (CFRP) composites. Such materials exhibit an excellent strength-to-weight ratio but have the drawback of low electrical conductivity due to the insulating polymer matrix imbedding the carbon fibers. This is a concern as airliners operating in rain and thunderstorms are exposed to a high risk of getting hit by a lightning strike, which happens about once every year for a commercial plane.^3,4^ A lightning strike can result in devastating damage, such as vaporization and pyrolysis of resin, delamination, fiber breakage, and thermal-burn damage when an insulating structural composite is hit. Indirect damage can occur once the electromagnetic interference (EMI) caused by a lightning strike affects or even breaks down advanced electrical systems and electronic equipment inside the aircraft.^4,5^ Composite materials hence require additional lightning strike protection (LSP) to avoid such damage.

The LSP solutions commonly used are based on metallic meshes attached to exterior composite parts of the aircraft. This LSP system provides the aircraft structure with high electrical conductivity, which enables the lightning current to flow with lower resistivity on top of the composite and exit toward grounded parts.^6^ The LSP’s endurance to EMI is referred to as EMI shielding effectiveness (EMI SE) and is shown to be related to the material characteristics, where a higher electrical conductivity enhances the EMI SE.^5,7,8^ Although the combination of the composite structure and metallic LSP is considerably lighter than the previous full metal structure, the metallic LSP still represents a significant weight gain for the aircraft (150–200 g.m^–2^). Novel lightweight LSP with preserved electrical conductivity has the potential to decrease CO_2_ emissions, as a lower weight is directly correlated to lower fuel consumption and a reduced environmental impact. In addition, the conventional metallic mesh combined with carbon fiber-reinforced composites can lead to corrosion, increasing maintenance and related costs.^9,10^

A promising alternative to the conventional LSP system is the use of conductive carbon-based nanomaterials. Graphene and its derivatives, carbon nanotubes (CNTs),^11^ graphite,^12^ reduced graphene oxide (rGO),^13,14^ and graphene nanoplatelets (GNPs)^15^ have all been successfully studied as conductive fillers due to their outstanding properties and are thus promising for the development of novel LSP systems. For aerospace applications, it is especially the electrical characteristic in combination with the low density of the nanomaterials that entails great interest. However, one of the major challenges in achieving a homogeneous dispersion of carbon nanofillers in an epoxy matrix is their tendency to form aggregates and restacks.^4,16^ The instability of the graphene dispersion is attributed to the delocalized π electrons. The stability can be improved by utilizing GO,^17^ however, to a cost of a reduced conductivity. The processing of the composites through ultrasonication of the carbon filled resin has also been shown to be effective in the production of homogeneous dispersions and thereby conductive nanocomposites.^15^ Another approach is to sterically counteract the aggregation and simultaneously form a potent conductive network by mixing CNTs and graphene derivatives^18−21^ for a synergistic effect.

These graphene derivatives have been used to produce reinforced CFRP composites for LSP applications by using various approaches. Most recently, Lin and colleagues evaluated the effect of CNT and carbon black on the lightning strike tolerance of CFRP samples and found that the nanoreinforced composites show significantly higher tolerance to lightning strikes.^22^ Kopsidas et al. used GNPs and CNTs for the same purpose and found that not only the nanoreinforced samples enhanced the damage tolerance, but they also performed similarly to the conventional copper mesh protection.^23^ Xia et al. employed silver-modified CNTs as conductive fillers and showed that just a single ply of reinforced CFRP can improve damage tolerance to lightning strikes, maintaining more compressive strength than the CFRP protected by conventional copper mesh.^24^ Other studies have shown similar results,^25^ and all of them attribute the damage tolerance improvement to a higher electrical conductivity of the composites. However, studies that evaluated the conductivity of nanoreinforced CFRPs have consistently found that the increase in electrical conductivity is either insignificant or quite modest.^26−30^ Therefore, the reason behind the effectiveness of CFRP composites containing conductive nanofillers is still unclear and requires further investigation.

**Figure 1:**
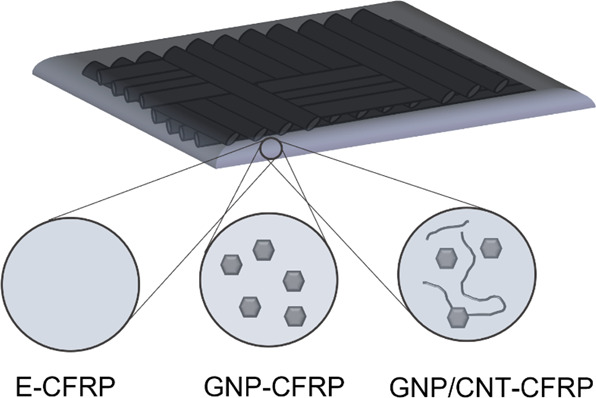
Schematic illustration of the three loading conditions used throughout this study: pristine epoxy (e-CFRP), epoxy loaded with 3 wt % GNP (GNP-CFRP), and an epoxy hybrid content of 0.5 wt % CNT and 2.5 wt % (GNP/CNT-CFRP).

The current work focuses on the examination of GNPs and CNTs as conductive additives in epoxy-based CFRPs and offers a framework to clarify the mechanism of damage reduction. For that, unlike most studies in the field cited above, this research offers a comprehensive conductivity characterization of both the reinforced epoxy nanocomposites and CFRPs produced with them. Initially, epoxy loaded with the nanofillers was evaluated, and three loading conditions were then chosen for producing single-ply CFRP samples (Figure 1) that were subjected to lightning strike simulation tests. Using single-ply CFRPs instead of stacking multiple layers allows for higher filler content compared to previous studies on hybrid GNP-CNT reinforcement.^23^ This is possible because the single-ply layer can serve solely as a protective outer layer, minimizing the stiffening effect of the nanoreinforcement on the mechanical properties of the entire panel. Finally, a simple, nondestructive method to assess the damage caused by the lightning strike test is proposed, using UV-illumination to reveal resin degradation on the surface. Ultrasonic c-scanning is widely used to effectively detect internal physical flaws (such as voids and delamination),^22,23^ but the UV-illumination method is able to reveal the extent of resin degradation on the surface and could be used as a complementary technique for damage assessment. The damage features are evaluated by microscopy, FTIR, and the proposed UV-illumination method.

## 2 Experimental Procedure

### 2.1 Materials and Preparation

#### 2.1.1 Materials

**Figure 2:**
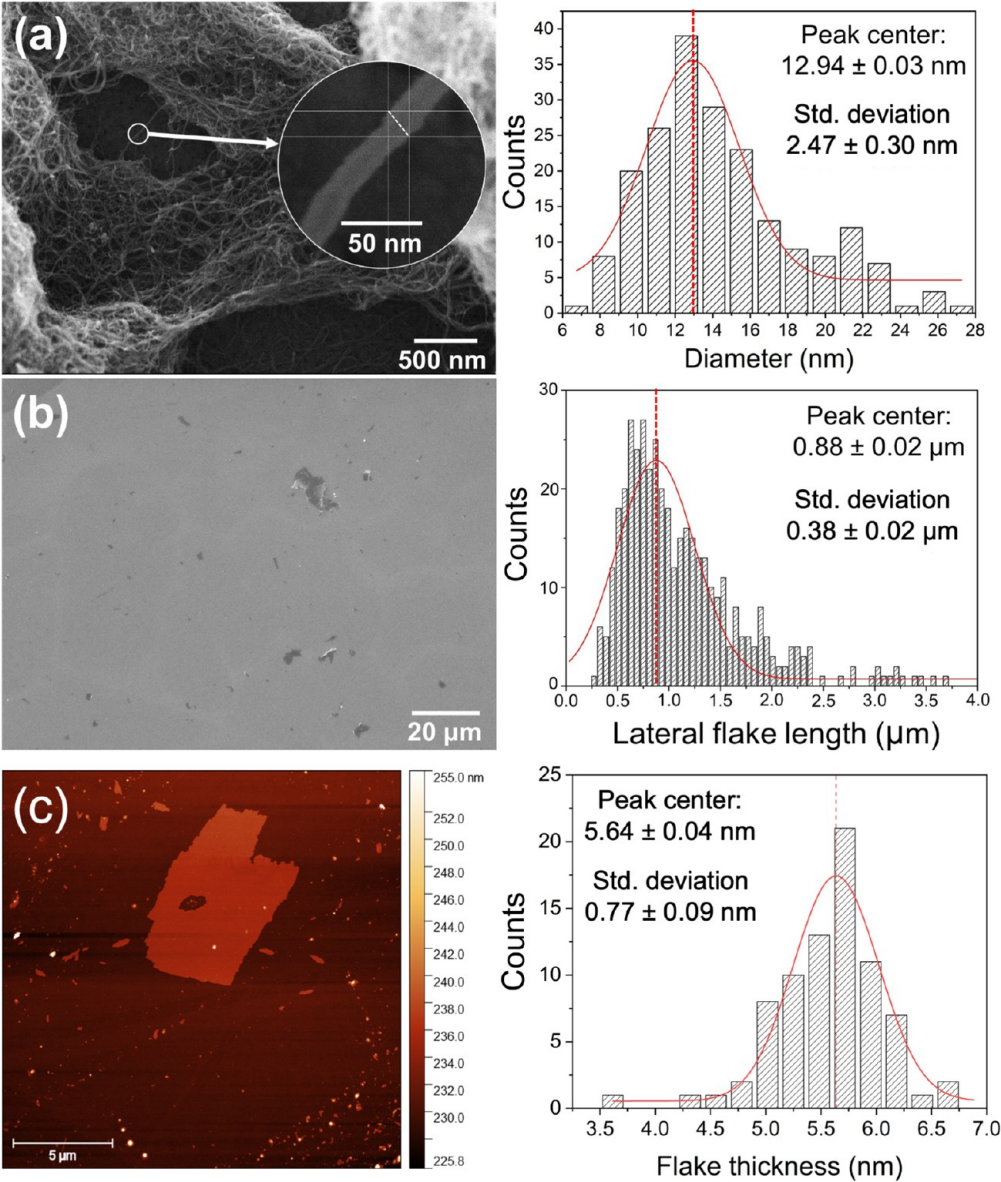
Representative SEM micrographs of the nanoparticles used, alongside histograms of the measured values of the CNT diameter (a) and GNP lateral size (b). Representative AFM micrograph of GNP flakes and their thickness distribution (c).

Aerospace-grade epoxy system Araldite LY 5052/Aradur 5052 was purchased from Huntsman. The woven carbon fiber fabric was purchased from FIBERMAX, Greece, and used as received. Graphene nanoplatelets were produced and provided by 2D fab and used as received. The flakes have an average thickness of 5.64 ± 0.77 nm, as measured by atomic force microscopy (AFM) (Bruker Dimension Icon, Massachusetts, USA), and the average lateral size is 0.88 ± 0.38 μm, as measured by scanning electron microscopy (SEM) (Jeol JCM-6000 Plus, Tokyo, Japan). Multiwalled carbon nanotubes NC7000 were purchased from Nanocyl, Belgium, and used as received. Average diameter of the tubes were measured by SEM to be 12.94 ± 2.47 nm (Figure 2).

#### 2.1.2 Preparation of GNP/CNT/Epoxy Systems

**Figure 3:**
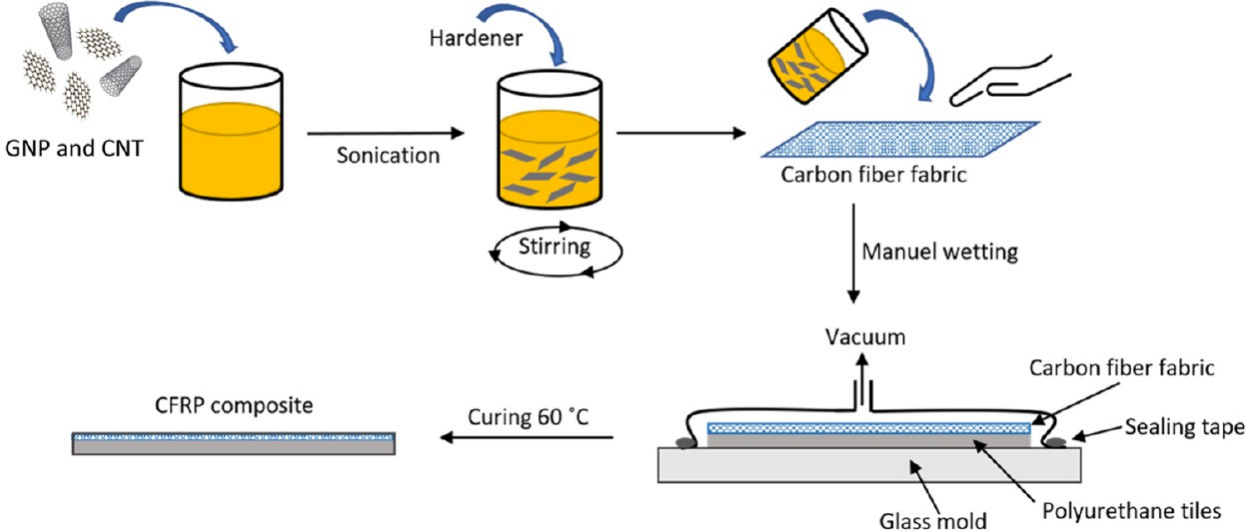
Schematic illustration of the preparation process for CFRP samples that underwent simulated lightning strike tests.

GNPs and CNTs were mixed directly into the epoxy resin at weight contents ranging from 0.1 to 7.5 wt % (GNP) and 0.01 to 1.0 wt % (CNT). A hybrid GNP 2.5 wt % + CNT 0.5 wt % sample was also prepared. The epoxy-nanofiller suspensions were dispersed for 2 h in an ultrasonic bath with a fixed frequency of 40 kHz (SoniClean 2PS, Sanders, Brazil). This method has proven to properly disperse these graphene- and hybrid-filled samples while maintaining the nanoparticles’ integrity.^15^ After sonication, the hardener was added and the mixture thoroughly stirred. For the nanocomposites without carbon fiber, the resulting slurry was poured into appropriate silicone molds and placed under vacuum for 5 min to remove bubbles, followed by a curing step of 24 h at room temperature and a postcuring step of 4 h at 100 °C. For the CFRP samples, single layers of woven carbon fibers were wetted manually with selected concentrations of nanoparticles/epoxy mixture and then applied on either a polyurethane tile (for lightning strike tests) (Figure 3) or a Teflon sheet (for conductivity measurements). Both panels were processed through vacuum-bagging and cured for 2 h at 60 °C. The resin:fiber ratio for all CFRP samples is close to 50:50.

### 2.2 Characterization

#### 2.2.1 Electrical Conductivity

Through-plane electrical conductivity was measured with a gain phase analyzer Solartron SI 1260, coupled with a Solartron 1296A dielectric interface. Dielectric spectra were taken from 0.1 Hz to 1 MHz, and AC conductivity (σ_AC_) was then calculated from the imaginary permittivity through eq 1:
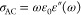
1where ω is the angular frequency, ε_0_ is the vacuum permittivity, and ε″ is the measured imaginary permittivity.^31^ AC conductivities are reported at the lowest frequency available (0.1 Hz). For nanofiller-reinforced epoxy samples without carbon fiber, disc-shaped specimens were prepared and coated with a 20 nm gold layer on both sides to remove contact resistance. For CFRP samples, discs were cut from the composite panels and also gold-coated on both sides. In-plane electrical conductivity of the CFRP composites was measured by the four-point probe method with a Keithley 2410 source meter, using rectangular specimens of ∼80 × 10 × 0.26 mm cut from the composite panels.

#### 2.2.2 Microstructure

The surface morphology of the CFRP was studied using a ZEISS LEO 1550 Scanning Electron Microscope (SEM), supplied with a Schottky field FEG, and arranged in a GEMINI column at an acceleration voltage of 3 kV. The nanofillers’ distribution inside the CFRP samples was investigated using an FEI Quanta 250 SEM in high-vacuum mode at 10 kV.

### 2.3 Lightning Strike Test and Damage Characterization

#### 2.3.1 Lightning Strike Test

The simulated lightning strike test was performed at the Ångström Laboratory of Uppsala University, Sweden. An impulse high-current generator ICG from Haefely AG, LtD., (Switzerland) produced a current waveform of *T*_1_/*T*_2_ = 10/350 μs. *T*_1_ represents the time required to raise the current from 10 to 90% of the peak, and *T*_2_ represents the time to raise the current from 10 up to 90% and then back to 50% of the peak.^13^ The action integral and charge transfer are defined as *I* = ∫ *i*^2^*dt* and *Q* = ∫ *idt* and correspond to the specific energy of the impulse current and the total energy, respectively, where *i* is the time-varying electrical current of lightning waveforms.^32^

**Figure 4:**
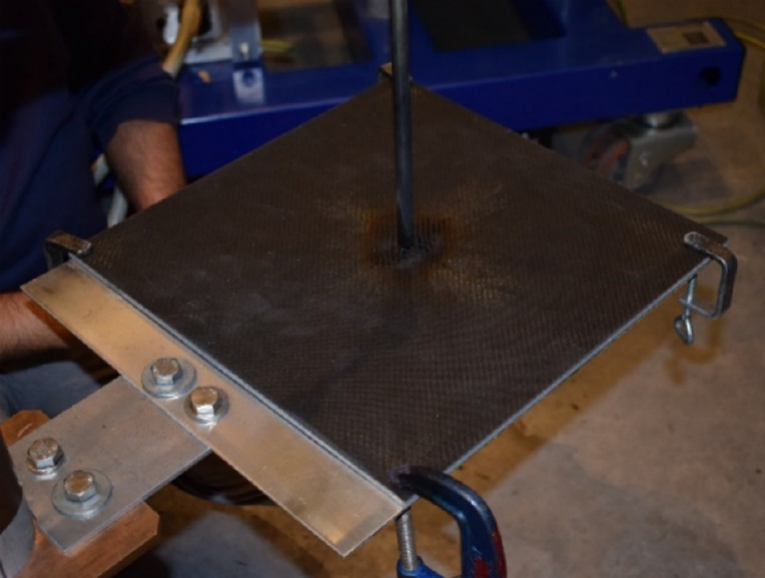
Experimental setup for CFRP specimens (picture taken after a simulated lightning strike).

The specimens were attached to a metal plate with four fasteners, one in each corner, and a discharge probe with a diameter of 7 mm was placed approximately 1.5 mm above the center of the specimen. The experimental setup for the lighting strike test can be seen in Figure 4.

#### 2.3.2 ATR-FTIR

Attenuated total reflectance Fourier transform infrared (ATR-FTIR) spectroscopy measurements were carried out with a PIKE MIRacle ATR accessory with a diamond prism in a Vertex 70 spectrometer with a DLaTGS detector. The whole system was continuously purged with nitrogen, and the IR spectra were acquired at 2 cm^–1^ resolution. A total of 64 scans were performed between 4500 and 600 cm^–1^. IR spectra were acquired from four areas of varying damage of the GNP/CNT-CFRP panel.

## 3 Results and Discussion

### 3.1 Electrical Conductivity of Carbon-Nanofiller-Loaded Epoxy Resin

Efficient lightning strike protection (LSP) is thought to require highly conductive materials, which can effectively transport charges toward grounded areas and avoid damage.^4,22^ Epoxy systems with different loads of GNPs and CNTs were then evaluated to investigate how the carbon additives affected the nanocomposites’ conductivity and find an optimal percentual loading.

**Figure 5:**
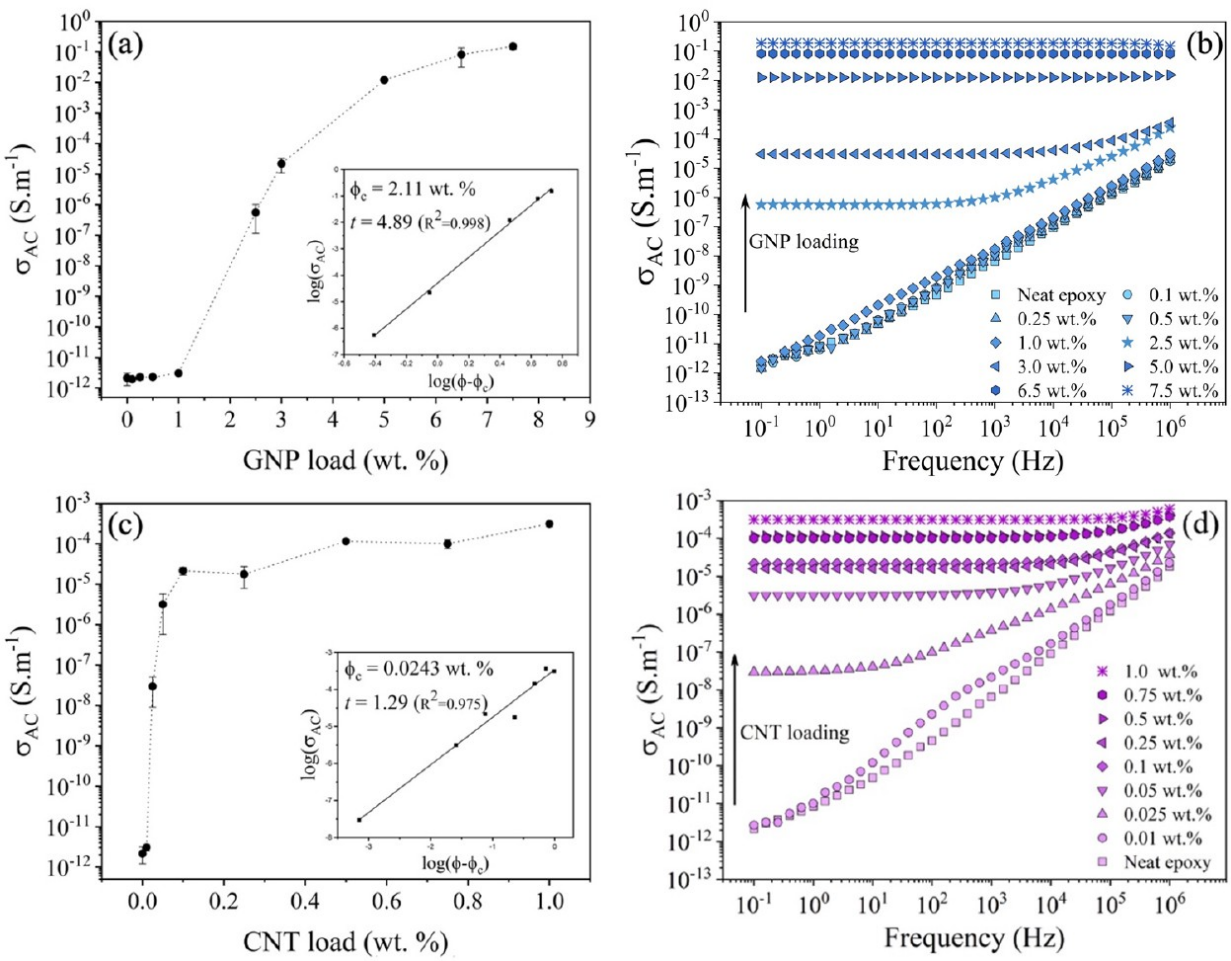
Electrical conductivity percolation curve of (a) GNP- and (c) CNT-epoxy nanocomposites; electrical conductivity spectra of (b) GNP- and (d) CNT-epoxy nanocomposites. Dotted lines are guides for the eye.

Figure 5a,b shows that the addition of GNPs decreased the inherently high electrical resistivity of epoxy. Although lower concentrations of up to 1 wt % displayed virtually no improvements, the conductivity increases rapidly from 2.5 wt % and reaches the value of 1.5 × 10^–1^ S·m^–1^ at 7.5 wt %, almost 11 orders of magnitude higher than neat epoxy. This sharp increase in conductivity is the hallmark of percolation behavior, which is common in systems that entail the introduction of conductive fillers into insulating matrices. The percolation threshold (PT) was calculated by fitting the curve using the power law:
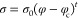
2where σ is the electrical conductivity, σ_0_ is a pre-exponential factor (dependent on the system), ϕ is the filler content (above ϕ_c_), ϕ_c_ is the critical concentration at the transition (PT), and *t* is the critical exponent.^33^ With this, the PT was calculated to be at 2.11 wt %.

Figure 5 b shows how electrical conductivity behaves as a function of frequency and gives further insight into the connectivity of the nanofiller network. When a system has not yet achieved electrical percolation, strong frequency-dependent behavior arises. Higher frequencies promote nonohmic conduction mechanisms, characterized by electron hopping and tunneling between GNP flakes that are separated by a thin, insulating matrix layer. As the connectivity of the network increases, a frequency-independent plateau is expected to appear as a sign that the conductive nanofiller particles are close enough to each other and ohmic conduction takes over.^34^ The spectra in Figure 5 b further show that the strong frequency-dependent behavior of samples with lower GNP loadings remains quite similar to that of neat epoxy, with only small shifts toward higher conductivities as concentration increases. At 2.5 wt %, however, there is not only a substantial increase in conductivity but also a clear transition to an almost frequency-independent behavior, providing evidence of the presence of an interconnected network. This agrees with the percolation threshold extracted from the power law (eq 2). Higher GNP concentrations only consolidate this behavior and further increase conductivity but now to a lesser degree. It is important to point out that, although samples loaded with more than 5 wt % achieved higher conductivities, the ensuing high viscosity (common to such high GNP concentrations) would pose a challenge for properly processing and preparing the composite material. Therefore, 3 wt % GNP was the chosen concentration for preparing the GNP-loaded CFRP panel.

CNTs are known to be effective nanofillers that drastically improve electrical conductivity in epoxy systems at low concentrations, not only due to their own high conductivity but also due to their high aspect ratio.^35−39^Figure 5 c,d shows that, at only 0.025 wt %, CNTs could improve conductivity over 4 orders of magnitude when compared to the insulating neat epoxy, achieving electrical percolation at 0.024 wt %. At this concentration, there is a clear shift from an entirely frequency-dependent behavior to a frequency-independent plateau at lower frequencies, denoting an insulator-to-conductor transition. Addition of CNTs continues to improve conductivity, but from 0.25 wt % on, it stabilizes around 10^–4^ S·m^–1^. This shows that, for the present system and dispersion method, CNTs are more efficient at creating an interconnected network, but the GNPs provided the nanocomposites with higher terminal conductivities when saturated.

**Figure 6:**
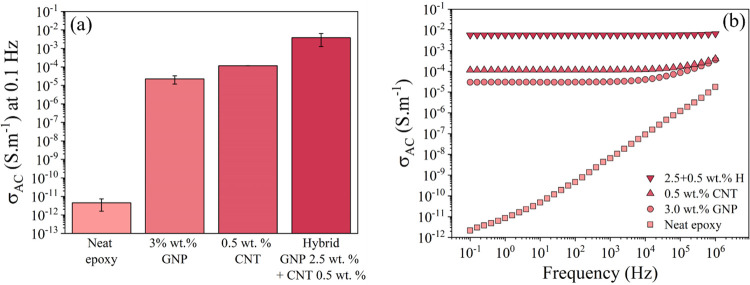
(a) Electrical conductivity and (b) electrical conductivity spectra of the different compositions of nanofiller-loaded epoxy resin used in the construction of CFRP panels.

To be directly comparable with the GNP-loaded composites, the concentration chosen for the hybrid-loaded samples was a total of 3 wt % of carbon nanoparticles, in which 2.5 wt % are GNPs (close to its percolation threshold) with the remaining 0.5 wt % of CNTs (above its percolation threshold). Figure 6 shows that the hybrid nanocomposite GNP/CNT/epoxy achieved a conductivity of 3.8 × 10^–3^ S·m^–1^, not only higher than 3 wt % GNP but also higher than 0.5 wt % CNT, which had already achieved the highest conductivity when only CNTs were added. This synergetic effect has been extensively discussed in the literature. The nanofillers’ different aspect ratios might prevent their restacking/reagglomeration after the dispersion process, and CNTs can also bridge the gap between adjacent GNP flakes.^19,21^ Our previous work on this subject showed that the combination of both nanoparticles decreased the amount of GNP and CNT agglomerations when processed by an ultrasonic bath.^15^

These results clearly show the relationship between the conductivity and carbon nanofiller loading. When applied in an aircraft structure, the resin is infused into carbon fiber fabrics to form a robust, mechanically stable composite material. To minimize the stiffening impact of the nanofillers on the mechanical behavior of the aircraft structure, a one-layer composite material was investigated. This layer could function as an additional LSP layer or substitute for the outermost layer in the composite, thereby reducing its impact on the overall mechanical properties of the fuselage. Three sample compositions were chosen for production of CFRP panels and further studies thereof: neat epoxy (e-CFRP), 3 wt % GNP (GNP-CFRP), and the hybrid 2.5 wt % GNP + 0.5 wt % CNT (GNP/CNT-CFRP). The 3 wt % loading was close to the identified GNP percolation threshold, the CNT/GNP was chosen due to the higher increase in conductivity, and neat epoxy was used as a reference. Carbon filler loads above 3 wt % in total were excluded due to the high viscosity and low processability.

### 3.2 Characterization of CFRP Composites

#### 3.2.1 Scanning Electron Microscopy (SEM)

**Figure 7:**
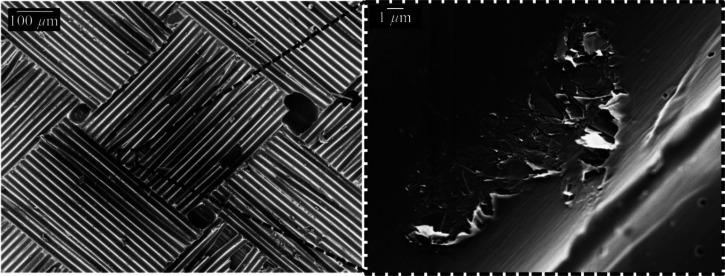
Top-view SEM micrographs of a GNP/CNT-CFRP panel.

Figure 7 shows a top-view image of a GNP/CNT-CFRP panel. The pattern from the peel-off fabric can be clearly seen in the overview image. In a higher magnification, a smooth surface is observed with GNP flakes in a size of about 10 μm visible occasionally. The CNT structures cannot be detected with this magnification. This indicates that the GNP flakes are embedded within the epoxy resin without being agglomerated at the surface.

**Figure 8:**
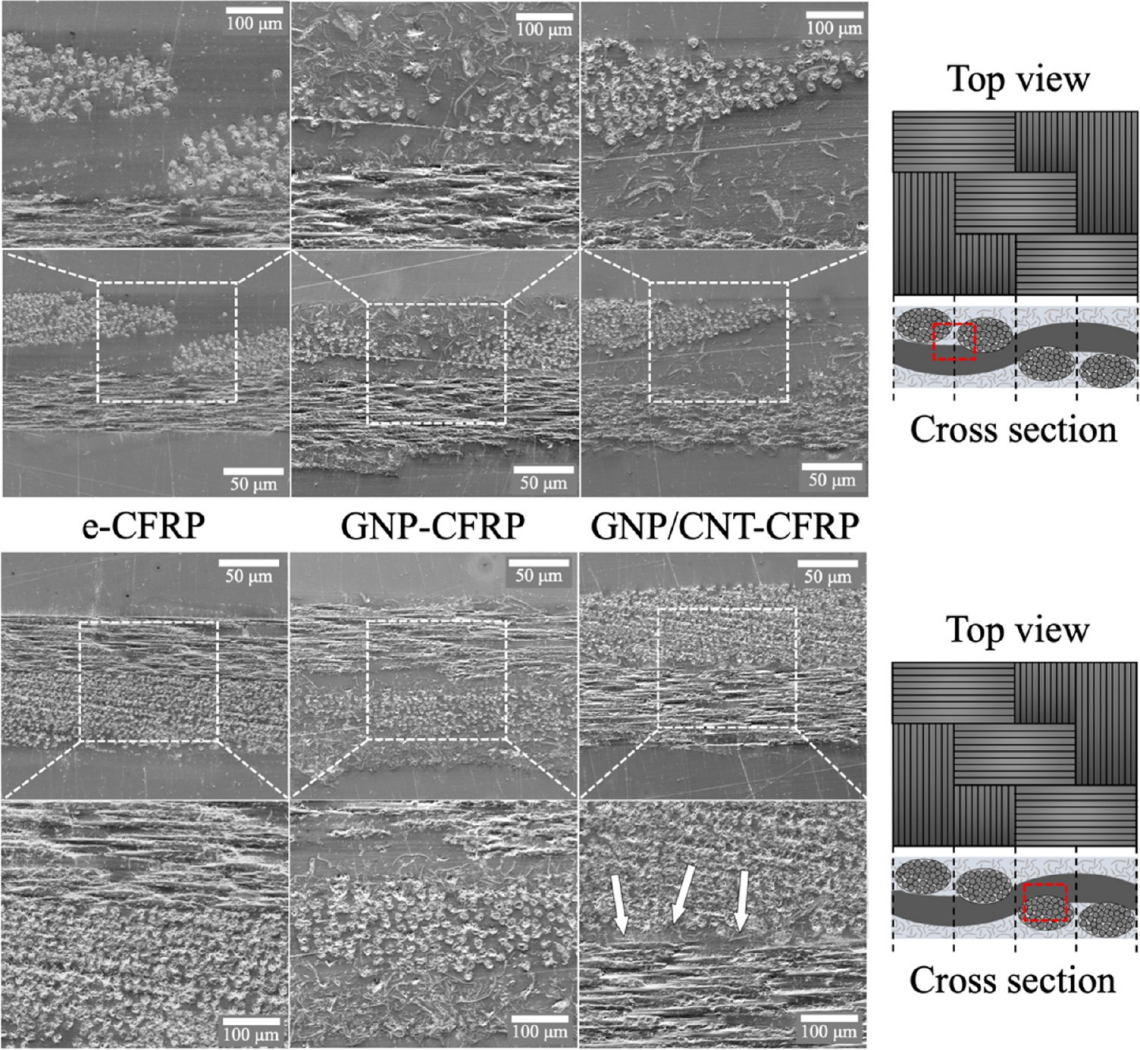
SEM micrographs of the CFRP panels’ cross sections made with neat epoxy, GNP-loaded, and hybrid GNP/CNT-loaded nanocomposites, from left to right, respectively. Upper micrographs display the region in which one carbon fiber thread runs between two perpendicular threads, clearly exposing the embedding resin. Lower micrographs show the region in which the gap between the bidirectional carbon fiber threads is minimal. The position of each region is illustrated in the schematics on the right-hand side. White arrows point to GNP flakes that are rather difficult to visualize.

To investigate the distribution of CNTs and GNPs further, SEM micrographs of the panels’ cross section were taken (Figure 8). The reinforced resin is observed in two different regions of the carbon fiber fabric pattern: the gap between two carbon fiber threads that run parallel to each other, in which resin is more exposed; and the most compact region between two perpendicular threads. Examining the GNP-CFRP panel and comparing it to the nonreinforced sample (e-CFPR), GNP flakes appear well distributed throughout the thickness of the composite. The flakes not only establish a conductive network in the outer layer of resin that envelops the carbon fibers but are also found embedded between the fiber threads. The extensive presence of conductive GNP flakes helps bridge the distance between the conductive carbon fibers that are separated by the insulating resin and, therefore, helps carry the high electric current from the lighting strike. A similar microstructure is seen in the hybrid GNP/CNT-CFRP panel, i.e., well-distributed GNP flakes throughout its thickness. Although there are fewer GNP flakes visible, it is important to consider that the GNP concentration in the hybrid-loaded sample is indeed lower than in the GNP-CFRP sample (2.5 instead of 3 wt %). CNTs could not be resolved with this magnification, but it is reasonable to expect that they were also successfully incorporated together with the GNPs.

#### 3.2.2 Damage Tolerance

The extent of damage in a one-layer, epoxy-infused carbon fiber textile was studied to evaluate the LSP protection ability. A one-layer composite LSP allows for light protection without jeopardizing the mechanical behavior of the structural material.

The damage tolerance of the three composite versions (e-CFRP, GNP-CFRP, and GNP/CNT-CFRP) was investigated after a simulated lightning strike test performed at 60,000 A. Direct damage from lightning strikes is generally divided into three modes, i.e., resin deterioration, fiber breakage, and delamination of composite plies.^4,32,40^ Resin deterioration includes pyrolysis, vaporization, and oxidation of epoxy, which could possibly result in a changed chemical structure of the epoxy resin.^32,40,41^

**Figure 9:**
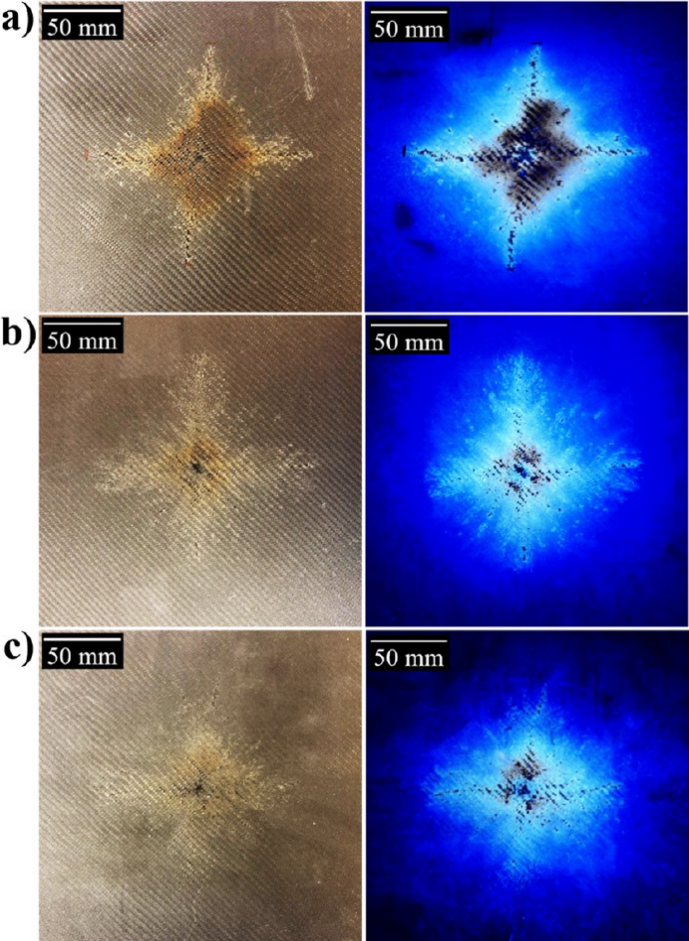
Composite panels of (a) e-CFRP, (b) GNP-CFRP, and (c) GNP/CNT-CFRP imaged in visible light and in UV light, respectively.

Figure 9 shows the CFRP composite panels of (a) e-CFRP, (b) GNP-CFRP, and (c) GNP/CNT-CFRP after the simulated lightning strike test. The damaged regions of all composites exhibit a similar shape, where the central area around the lightning attachment point displays resin burn and fiber breakage. From the central point, all images show a rhombus shape of possible resin deterioration with a cross-like form of pyrolysis and fiber damage, which agrees with previous studies.^32,40−42^ The studies by Li et al.^21^ and Hirano et al.^32,40^ showed the same rhombus structure as that in this work but with more extended damage in one direction.

**Figure 10:**
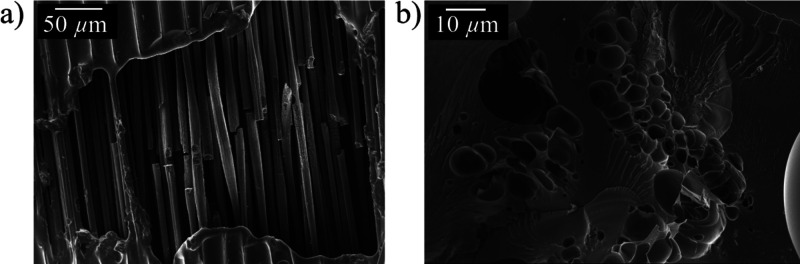
CFRP after lightning strike test observed by SEM: (a) area of resin deterioration; (b) resin deterioration process.

This phenomenon might be due to the organization of the fibers. In previous studies, unidirectional prepreg systems were used where different stacking sequences could result in damage varying in different directions. In the present study, the fibers are woven with an angle of 90° where the cross-like fiber damage follows the two fiber directions to a similar extent. In addition, the high thermal conductivity of carbon fibers might help to explain the cross-shaped lightning damage on CFRP. A work by Wang et al.^43^ revealed that the large temperature increase caused by the lightning distributes over the composite structure in the fiber direction, generating heat concentrated paths. These paths receive more extensive damage as the resin burns, pyrolyzes, and deteriorates due to heat. Figure 10 shows SEM images of the damaged CFRP structure after the lightning strike test. In panel a, resin pyrolysis and deterioration are evident both on the surface and between the carbon fibers. Meanwhile, carbon fibers have started to break, which makes the composite structure vulnerable and seriously damaged. Figure 10b shows a bubble-like structure possibly due to voids forming after resin vaporization.

Previous studies show severe delamination between the CFRP plies close to the center of the damage.^32,43^ A hypothesis is that the pyrolysis and burning of resin could result in entrapment of gas in between the layers resulting in an explosion-like fracture.^32^ This phenomenon is not observed in this study. A one-layer woven system could possibly reduce the entrapment within the layers, and delamination is thereby avoided. Also, the insulating polyurethane tile utilized underneath the CFRP layer could possibly reduce the ballistic effect from the explosion-like failure.

The neat epoxy (e-CFRP) clearly reveals a wider damage area than the reinforced composite panels (Figure 9) as the pyrolysis, fiber breakage, and delamination in the center are more extensive. Possible resin deterioration and oxidization are more challenging to identify with the bare eye in visible light as they affect the molecular structure of the resin rather than forming visible burns.

**Figure 11:**
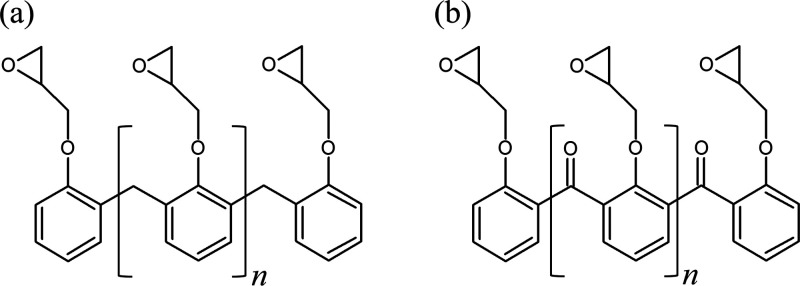
Araldite LY5052 epoxy novolac resin (a) and diphenyl ketone (b) formed after thermally induced oxidation.

The chemical structure of Araldite LY5052, a Novolac epoxy resin used in this study, is shown in Figure 10a. The deterioration of the epoxy following a lightning strike most probably results in degradation, oxidation, and changes in the chemical structure of the epoxy material. Pei et al.^44^ suggested in a previous study that the thermal degradation and oxidation of epoxy may result in oxidation of the methylene group of the epoxy molecule, forming diphenyl ketones (Figure 11b). Diphenyl ketone absorbs light in the region of about 360 nm, while it exhibits fluorescence in the visible region between 400 and 500 nm.^45^ The resin deterioration area after the simulated lightning strike was hence visually inspected during irradiation with UV light to evaluate possible diphenyl ketone formation (Figure 9, right).

A clear fluorescent area is visible around the center of the lightning strike. This may appear due to the resin degradation where the methylene groups are oxidized to diphenyl ketone.^44^ The intensity of the fluorescence becomes stronger close to the lightning strike and decreases with the distance from the center. This could be because a lower extent of the resin is oxidized at a longer distance from the center, resulting in a lower fluorescence intensity. In the black area in the middle, the epoxy is completely pyrolyzed and burnt, and the carbon fibers are exposed. The results clearly show that the resin deterioration is more extensive in the neat epoxy, while the GNP and CNT-loaded composites have a higher resistance toward resin degradation. The image also indicates that the combined loading (GNP/CNT-CFRP) outperforms the version in which only GNPs are added.

To investigate the difference in the deterioration area further, image binarization and the software ImageJ 2.0 were used to estimate the size of the damaged areas from the fluorescent images (Supporting Information, Figure S1).^13,32^ From this, the size of the damaged area in the neat epoxy is measured to be 286 cm^2^ while the same area for the GNP-CFRP composite is 269 cm^2^ (Figure S2). The deterioration area of the hybrid composite (GNP/CNT-CFRP) is approximately 186 cm^2^. The difference in the size of the damaged area further shows the advantage conferred by the hybrid loading. The results indicate that the GNP loading reduces the burn, while the concept of hybrid loading reduces the deterioration damage significantly.

**Figure 12:**
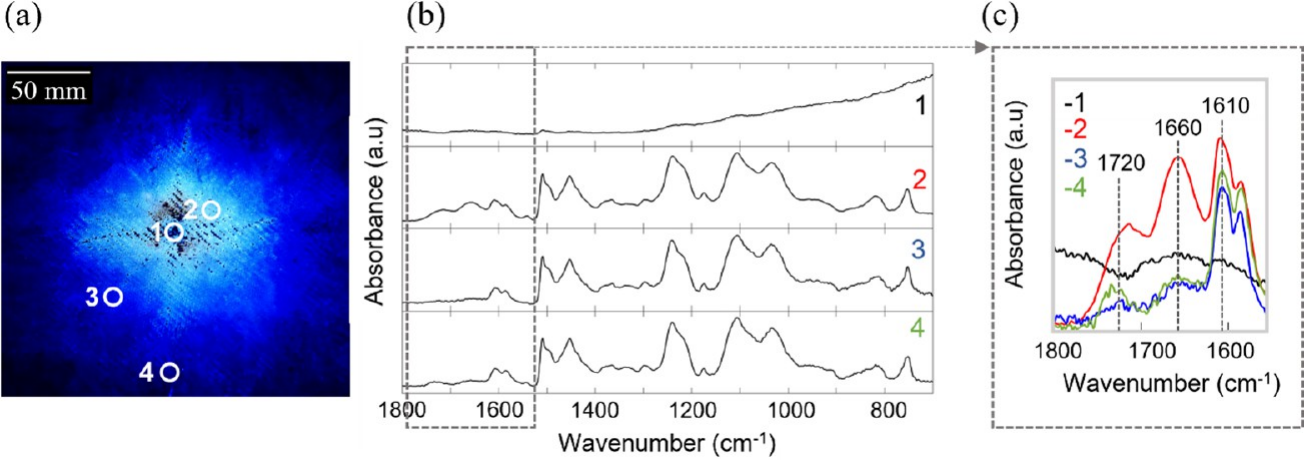
Position of areas 1–4 in the damaged GNP/CNT-CFRP sample (a). ATR-FTIR overview spectra of the fingerprint region of the different areas (b) and an enlargement of the range between 1550 and 1880 cm^–1^ (c). The full scan between 4500 and 600 cm^–1^ can be found in Figure S3.

Four different areas of the GNP/CNT-CFRP composite were studied through ATR-FTIR to further investigate the possible change in the chemical structure of the epoxy upon lightning strike damage (Figure 12). In the center of the damage, epoxy was completely pyrolyzed and no ATR-FTIR signal is observed. It is important to note that the lack of signal in this pyrolyzed area might be a limitation of a diamond-based ATR-FTIR technique, as carbon has a higher refractive index than diamond. In regions 2–4, most of the bands and their relative heights in the fingerprint area (Figure 12b) within the region between 1800 and 700 cm^–1^ remains unchanged. However, significant differences are observed in the region between 1800 and 1550 cm^–1^ (Figure 12c). At a longer distance from the center (areas 3 and 4), the bands’ heights and relative ratio are similar, but at a closer distance to the center (position 2), in the clear blue, fluorescent area, two bands arise. The band at about 1660 cm^–1^ is attributed to stretching vibration of C=O in a diphenyl ketone, while the band at about 1720 cm^–1^ is attributed to the ketone group. The slight decrease in the 2920–2850 cm^–1^ bands (Figure S3) suggests the consumption of methylene groups, further supporting the diphenyl ketone formation proposed by Pei et al.^44^ This is a clear indication that the epoxy is oxidized, most probably due to heat development during the test. It also shows the possibility of using UV light as a powerful detection tool for investigating heat damage and resin deterioration on the surface, complementing other in-depth structural inspection techniques such as ultrasonic c-scanning.

#### 3.2.3 Electrical Conductivity of the CFRP Composites

**Figure 13:**
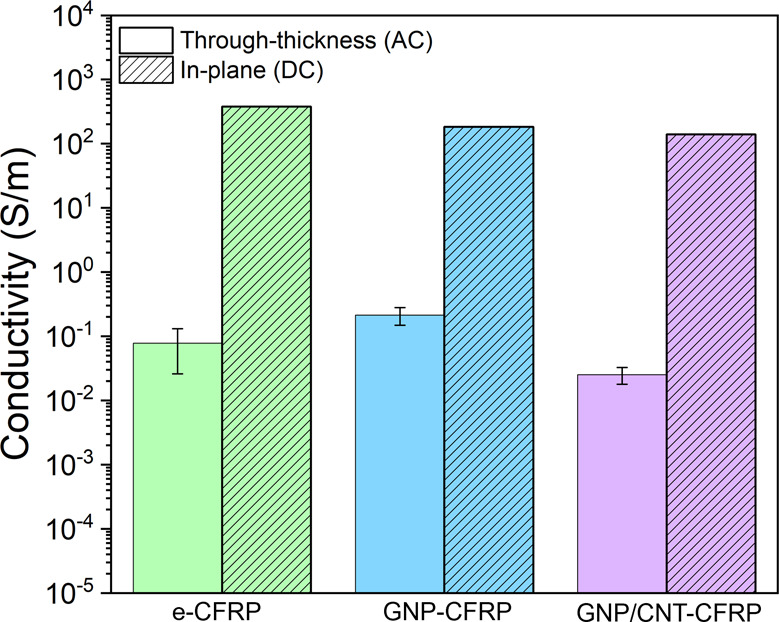
Electrical conductivity of all the CFRP panels prepared, measured in the through-thickness and in-plane directions.

Electrical conductivity tests were performed on the CFRP samples to assess whether the significant damage reduction shown by the reinforced composite panels after lightning strike tests was related to a gain in conductivity. As discussed previously, the heat generated by the Joule effect plays a major role in the damage mechanism and it is given by the widely known Joule’s law (eq 3):
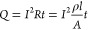
3in which *Q* is the amount of heat generated, *I* is the current, *R* is the resistance, *t* is the duration of current flow, ρ is the electrical resistivity, *l* is the length, and *A* is the cross-sectional area of the material. The higher the resistance, the more heat is generated, and therefore, decreasing the material’s electrical resistivity ρ is indeed a direct way to decrease joule heating degradation. Also, as the enhancement in electrical conductivity of the epoxy resin was clearly seen after loading it with the carbon-based nanomaterials, it was expected that the many orders of magnitude improvement on the matrix would translate into higher conductivities for the CFRP panels. However, as seen in Figure 13, the through-plane conductivity is only slightly higher in the GNP-CFRP sample, while no improvement was seen in the hybrid GNP/CNT-CFRP when compared to the nonreinforced panel (e-CFRP). The in-plane conductivity of the panels is much higher since it is measured in the direction of the conductive carbon fibers, but there are no significant changes among the three conditions.

Although counterintuitive, this result agrees with those in previous works. Robert et al.^26^ prepared CNT-reinforced CFRP panels, and the electrical conductivity increased less than an order of magnitude for 0.25 and 0.5 wt % loadings. When the CNT loading increased to 0.75 wt %, there was a decrease in conductivity when compared to the other reinforced panels, which the authors attributed to defects and bubbles caused by high viscosity. Dong et al.^27^ introduced 1.0 and 2.5 wt % of CNTs in the CFRP composite by immersing the carbon fiber in a CNT aqueous solution. Even at such high weight content, both samples showed less than 1 order of magnitude improvements. Bekyarova and colleagues^28^ also investigated the deposition of 0.25 wt % of single- and multiwalled CNTs on carbon fiber before resin impregnation. Again, less than an order of magnitude improvement in electrical conductivity was achieved for both CNT types in the out-of-plane direction. For the in-plane direction, a slight decrease in conductivity was observed for the single-walled CNT sample when compared to the neat epoxy CFRP. For GNP-filled samples, similar slight improvements in conductivity are also reported.^29,30^ The reason for such a modest or nonexisting increase in electrical conductivity seems to lie in the fact that the medium in which the nanofillers are dispersed (i.e., epoxy with carbon fiber embedded) is already a somewhat conductive material. At around ∼50 wt %, the conductive carbon fibers’ percolation has already been achieved and the addition of nanofillers cannot increase the CFRP’s conductivity much further. An interesting comparison can be made with glass fiber reinforced polymers (GFRPs) that, unlike CFRPs, are insulating materials. Studies that incorporated conductive carbon fillers in GFRPs realized improvements of many orders of magnitude in electrical conductivity,^46,47^ which could only be achieved due to the high resistivity of the starting GFRP medium.

**Figure 14:**
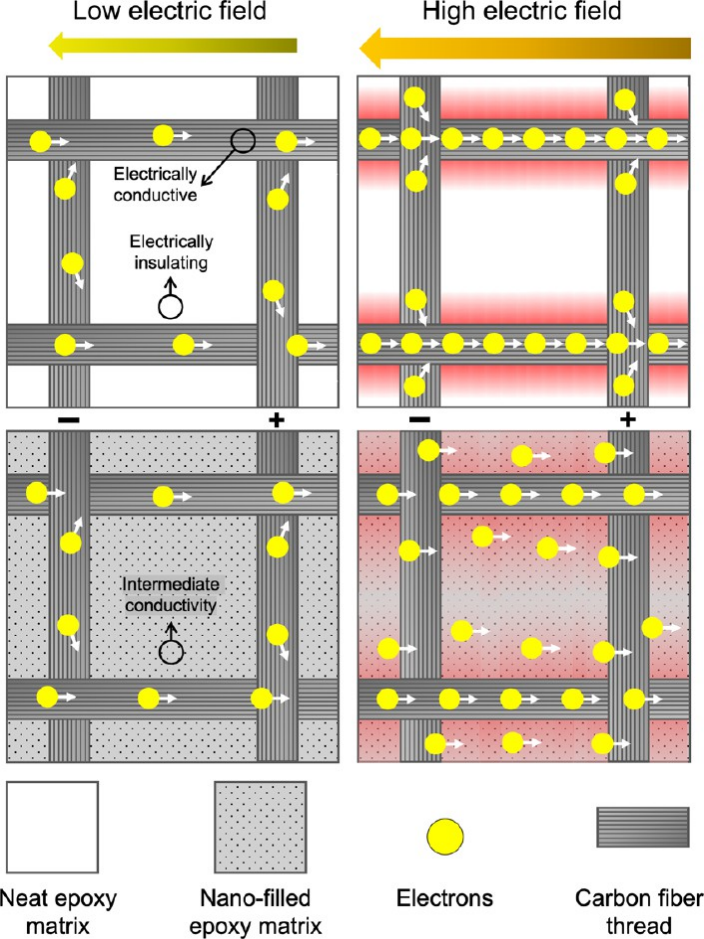
Illustration of the proposed mechanism for the CFRPs’ response at different voltage levels. At low voltage, charges travel primarily through the highly conductive carbon fibers, even with the improved conductivity of the nanofilled epoxy matrix over neat epoxy. During the lightning strike simulation, however, the electric fields are high enough to drive some current through the nanofilled matrix, spreading out the current and decreasing localized heat around the fibers when compared to the nonreinforced matrix.

Nevertheless, it is crucial to understand how the nanofillers improved the damage tolerance of CFRP samples in lightning strike simulations without simultaneously enhancing their overall conductivity. This discrepancy can be attributed to the different voltage levels to which the material is responding in each case. At the low voltage of the impedance spectroscopy test performed to assess the materials’ resistivity (3 V_RMS_), the voltage drop between an energized fiber and its adjacent fibers might not be high enough to drive current through the matrix between them. Thus, current flows primarily through the carbon fibers, which are still far more conductive than the nanofilled epoxy matrix,^48^ and long-range conduction occurring primarily through the points of contact between fibers. Therefore, as discussed earlier, the presence of the nanofillers in the matrix does not contribute significantly to the overall conductivity. However, in the lightning strike simulation, the electric fields that emerge are sufficiently high to drive current from fiber to fiber even through the nanofilled matrix. In this case, improvement in the conductivity of the matrix makes a significant contribution. This leads to a dispersion of current, akin to increasing the cross-sectional area in eq 3 whereby less heat is generated, contributing to the observed reduction in damage. This mechanism is illustrated in Figure 14.

This hypothesis is supported by the observable change in the damaged area’s shape, from a cross-shaped pattern in the e-CFRP sample to a more rounded area for the nanofiller-reinforced panels (Figure 9). In the absence of nanofillers, the current from the lightning strike is constrained to flow through the only conductive paths that are available, i.e., the carbon fibers within the e-CFRP sample^48^ woven at a 90° angle. The fibers carry virtually all the current and degrade the resin in their vicinity due to the extreme temperatures generated by Joule heating. This results in a distinctive cross-shaped damage pattern. For nanofilled samples, however, the presence of GNP and CNT under high voltages enables current to flow in radial directions that were not available before, leaving behind a rounder and less damaged area.

The better performance of the GNP/CNT-CFRP sample over the GNP-CFRP system in preventing damage is then related to the higher conductivity of the epoxy matrix when filled with the hybrid nanofillers. Another factor that could also play a role in this phenomenon is the current density. Previous works have demonstrated that CNTs can withstand current densities around a hundred times higher than graphene: 10^9^–10^10^ versus 10^7^–10^8^ A.cm^–2^, respectively.^49−52^ Therefore, it is possible that the CNT networks in the hybrid nanocomposite were able to withstand higher current densities before failure than did the GNP networks, improving the overall lightning strike tolerance.

## 4 Conclusions

The lightning strike damage tolerance and effect of carbon-based nanofillers were studied. A one-layer of pristine carbon fiber-reinforced plastic (e-CFRP) and nanocomposites filled with either 3 wt % graphene nanoplatelets (GNP-CFRP) or a combination of 2.5 wt % graphene nanoplatelets and 0.5 wt % carbon nanotubes (GNP/CNT-CFRP) were compared. It is concluded that

1. The electrical conductivity of the epoxy matrix is clearly improved through the addition of carbon-based nanofillers. The percolation of graphene-filled epoxy is reached at a level of 2.11 wt % GNP. A combination of GNP and CNT fillers is shown to further improve the conductivity of the epoxy matrix, most probably due to their difference in aspect ratio preventing agglomerations. Even though the conductivity of the matrix itself is clearly increased through addition of nanofillers, no significant improvement in conductivity between the different CFRP composites is observed.
2. The damage tolerance is clearly enhanced with addition of carbon-based nanofillers. The hybrid filling (GNP/CNT-CFRP) shows the best tolerance with a clear reduction in pyrolysis and resin deterioration. This can be attributed to the nanofillers’ network, which effectively spread out the current in radial directions.
3. The improved lightning strike tolerance in the absence of a significant enhancement in overall conductivity of the CFRP samples is discussed for the first time. This is attributed to the materials’ distinct response under different voltage levels, which make it difficult to predict the effectiveness of the nanoreinforcement only by standard conductivity measurements.
4. Resin deterioration is efficiently detected through the proposed UV-illumination method, proving its value as a complementary technique for damage assessment. The heat-induced resin deterioration following a lightning strike results in oxidation of the epoxy system, forming fluorescent diphenyl ketone entities. The degradation could also be detected through ATR-FTIR.

## Supporting Information Available

Binarized images of the UV-illuminated damaged areas, damaged area size measurements, and full FTIR spectra (PDF)
