# Supplementary material for: Hybrid Nanofiller-Enhanced Carbon Fiber-Reinforced Polymer Composites (CFRP) for Lightning Strike Protection (LSP)
Source: ACS Omega. 2024 Aug 9;9(33):35567–78. doi: 10.1021/acsomega.4c03272 (PMC11339985; doi:10.1021/acsomega.4c03272)
Supplement: Supplementary file 1 — ao4c03272_si_001.pdf [file ao4c03272_si_001.pdf]

Supplementary Material

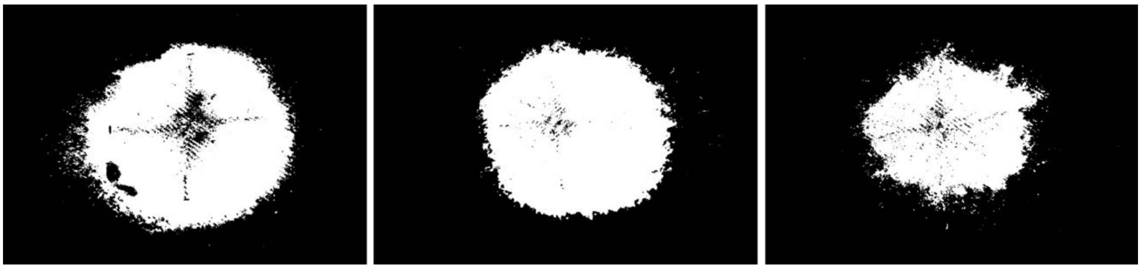

Figure S1. Imagine binarization from the fluorescent images done in ImageJ 2.0 freeware, which were used to estimate the size of the damaged areas. From left to right: e-CFRP, GNP-CFRP and GNP/CNT-CFRP.

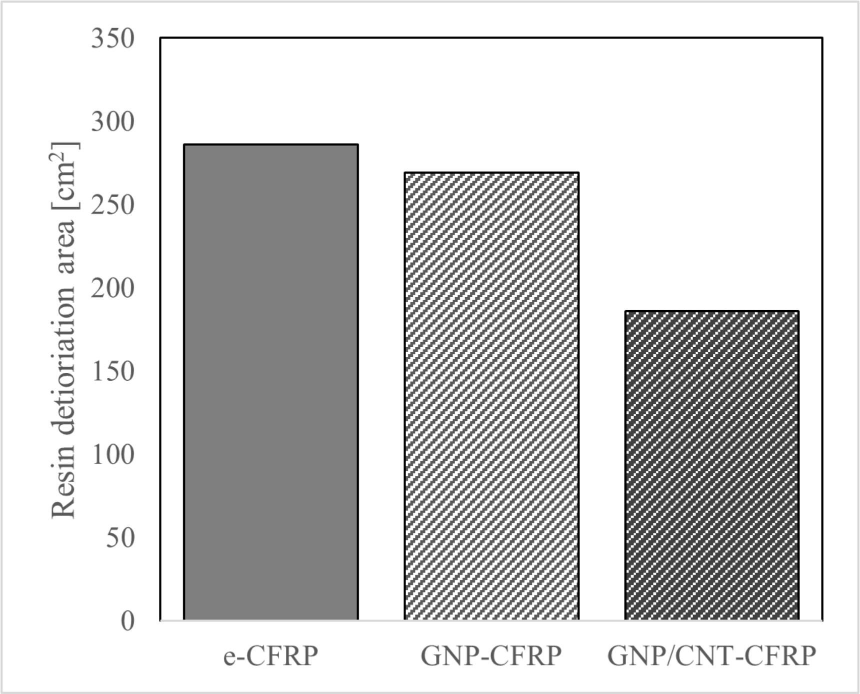

Figure S2. Size of the damaged areas after lightning strike simulation for each of the samples tested.

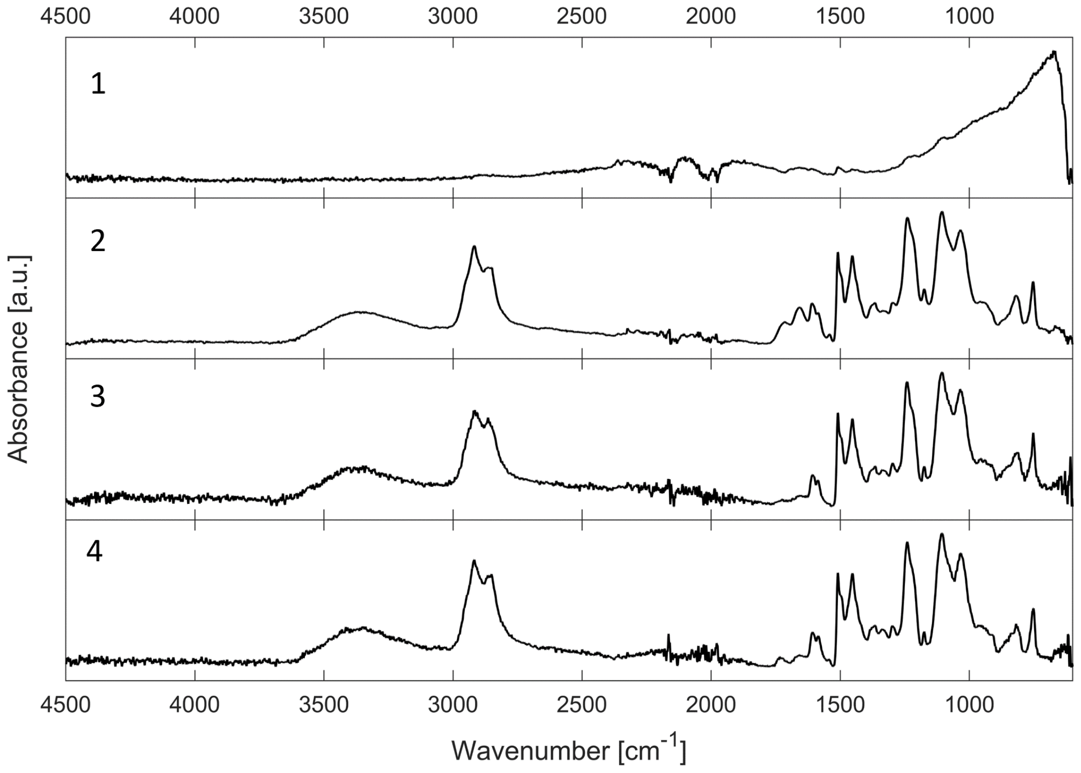

Figure S3. Full FTIR scan of the different regions of the damaged area after a lightning strike simulation (refer to Figure 11(a) on the main text).
